# Osteoarticular Infections in Immunocompetent Children Due to Atypical, Fastidious or Unusual Bacterial Pathogens: A Review

**DOI:** 10.3390/pathogens15060649

**Published:** 2026-06-19

**Authors:** Ardian Ramadani, Giacomo de Marco, Oscar Vazquez, Elio Paris, Andreas Tsoupras, Christina Steiger, Romain Dayer, Dimitri Ceroni

**Affiliations:** 1 Paediatric Orthopedics Unit, Geneva University Hospitals and University of Geneva, CH-1205 Geneva, Switzerland; ardian.ramadani@hug.ch (A.R.);; 2 Faculty of Medicine, University of Geneva, CH-1211 Geneva, Switzerland

**Keywords:** osteoarticular infection, osteomyelitis, septic arthritis, atypical pathogens, fastidious organisms, nucleic acid amplification testing

## Abstract

Osteoarticular infections (OAIs) present a significant diagnostic and therapeutic challenge to paediatric clinical practice. When evaluating suspected paediatric OAIs, the principal pathogens commonly considered are *Staphylococcus aureus*, *Streptococcus pyogenes*, *Streptococcus pneumoniae* and *Kingella kingae*. However, advances in molecular diagnostic techniques, particularly polymerase chain reaction assays and next-generation sequencing, have considerably improved the detection of less commonly identified pathogens responsible for OAIs. Consequently, familiarity with fastidious, emerging, exposure-related and uncommon bacterial pathogens is essential to ensure accurate diagnoses and appropriate therapeutic interventions. This review summarises bacterial pathogens responsible for OAIs in immunocompetent children that are fastidious, emerging, exposure-related or otherwise less commonly encountered. We describe their microbiological characteristics, clinical phenotypes, diagnostic pitfalls and organism-specific diagnostic strategies.

## 1. Introduction

Paediatric osteoarticular infections (OAIs) are major diagnostic and therapeutic challenges as they can lead to prolonged morbidity, systemic complications, joint damage, growth disturbances and long-term functional impairment. Accurate identification of the causative organism is central to its effective management, as it confirms the microbiological diagnosis, guides targeted antimicrobial therapy and may improve clinical outcomes. Furthermore, precise pathogen identification enables detailed assessment of the antibiotic resistance profile, which is essential for guiding antimicrobial therapy.

Before the early 2000s, pathogen detection depended mainly on traditional cultures of blood, joint fluid or bone aspirates [1,2,3]. However, despite the appropriate sampling of paediatric OAIs, culture-negative results were common: 24–68% of cases of acute haematogenous osteomyelitis and 21–70% of cases of septic arthritis [1,2,3]. These culture-negative infections may have reflected the presence of fastidious organisms, low bacterial inoculum, prior antibiotic exposure or the intrinsic limitations of conventional culture-based methods. Over the past 20 years, the greater sensitivity and specificity of nucleic acid amplification assays (NAAAs) have greatly enhanced pathogen detection in cases of paediatric OAI. Beyond pathogen identification, some NAAAs can detect genes associated with antibiotic resistance mechanisms and virulence factors [4]. Next-generation sequencing represents the most advanced iteration of NAAA technology. As microbiology progressively shifts from phenotype-based approaches toward genomic characterization, these techniques may become increasingly important for paediatric. Plasma-based, metagenomic, next-generation sequencing is a particularly promising approach, as it may be able to detect the pathogens responsible for localised infections from circulating microbial DNA [5,6,7]. Taken together, these advances suggest that pathogen identification and resistance profiling may increasingly become integrated into the routine for paediatric OAIs. Nevertheless, important limitations remain, including restricted availability, cost, limited standardisation and the potential difficulty of interpreting positive molecular findings in the absence of clear clinical correlation. Furthermore, molecular results should always be interpreted within the appropriate clinical and microbiological context, as contamination, colonisation or detection of non-viable organisms may occasionally complicate interpretation. Consequently, molecular techniques should currently be considered complementary rather than complete replacements for conventional microbiological methods.

Clinicians are therefore increasingly likely to encounter a broader spectrum of uncommon, fastidious and emerging bacterial pathogens in community-acquired OAIs affecting otherwise healthy children. Due to clinicians’ limited experience with these atypical and rarely occurring pathogens, a heightened awareness of these infections is essential for accurately identifying and effectively managing them. This review examines bacterial pathogens responsible for paediatric OAIs in immunocompetent children, that are fastidious, emerging, exposure-related, culture-negative, or otherwise atypical from a diagnostic or clinical perspective, with an emphasis on their microbiological features, epidemiology, clinical phenotypes and diagnostic approaches. For clarity, pathogens discussed in this review are grouped according to their predominant microbiological characteristics, epidemiological context, diagnostic challenges and degree of clinical recognition, acknowledging that overlap between categories may exist.

## 2. Literature Search

This review was based on a structured literature search performed in PubMed, Embase and the Cochrane Library. The search terms covered studies published between January 1995 and December 2025 and combined terms related to paediatric osteoarticular infections (including “paediatric”, “children”, “osteomyelitis”, “septic arthritis”, “osteoarticular infection”) with terms related to uncommon pathogens (including “fastidious”, “atypical”, “uncommon”, “emerging”, and specific pathogen names). Reference lists of relevant articles were also manually screened to identify additional studies.

Studies eligible for inclusion included original clinical investigations, cohort studies, case series, and selected case reports addressing epidemiological, microbiological, diagnostic, or therapeutic aspects of osteoarticular infections caused by fastidious, emerging, exposure-related, culture-negative, or otherwise atypical bacterial pathogens in immunocompetent children. Priority was given to paediatric studies and clinically relevant microbiological reports. Animal studies, in vitro studies, conference abstracts, instructional lectures and publications without sufficient clinical data were excluded.

## 3. Microbiological Basis: Fastidious & Atypical Organisms

Fastidious microorganisms have complex or specific nutritional needs and will only grow when certain nutrients are present in their environment: they are thus difficult to cultivate using standard methods. Nevertheless, the concept of fastidiousness extends well beyond the simple notion of dependency on specific nutrients. It includes the requirement for a variety of chemical signals, many of which are produced by surrounding bacterial species. As early as 1974, Lewis Thomas noted in *The Lives of a Cell* [8] that most microbes could not be cultivated alone because they exist in dense, interdependent communities reliant on complex chemical signals. This insight highlighted the limitations of the classic culture-based methods used to detect certain microorganisms.

Atypical bacteria are prokaryotes that differ from standard bacteria due to features such as absent or atypical cell walls, uniquely specialised growth requirements or an obligate intracellular mode of replication. Conventional Gram staining may poorly visualise or inconsistently characterise them, for the same reasons. This group typically includes organisms such as *Mycoplasma* spp., which lack cell walls, *Chlamydia* spp., which are obligate intracellular pathogens, and *Legionella* spp., which require specialised culture conditions [9,10,11]. Although uncommon, atypical and fastidious bacteria have been increasingly recognised as potential causes of OAIs, particularly when conventional cultures are negative. Diagnoses often depend on molecular methods instead of routine cultures because these bacteria have strict growth requirements and may be intracellular [10,11].

A growing body of case reports and small series studies has identified a diverse array of fastidious and atypical microorganisms involved in paediatric OAIs, many of which were only identified thanks to extended culture techniques or molecular diagnostics. These include HACEK group microorganisms, anaerobes, slow-growing Gram-negative bacilli and atypical bacteria, all characterised by their low or negligible culture yields using conventional methods. Knowledge of the epidemiological and clinical characteristics of these less common pathogens is becoming increasingly important since current molecular diagnostic techniques will identify them more frequently. Clinicians managing paediatric OAIs will be increasingly likely to encounter uncommon pathogens for which routine orthopaedic and paediatric practice provides limited guidance. The main fastidious, emerging, exposure-related and rare bacterial pathogens associated with paediatric OAIs are summarised in Table 1. For clarity, pathogens are grouped according to their predominant microbiological, epidemiological and clinical context, while acknowledging that overlap between categories may exist.

**Figure 1:**
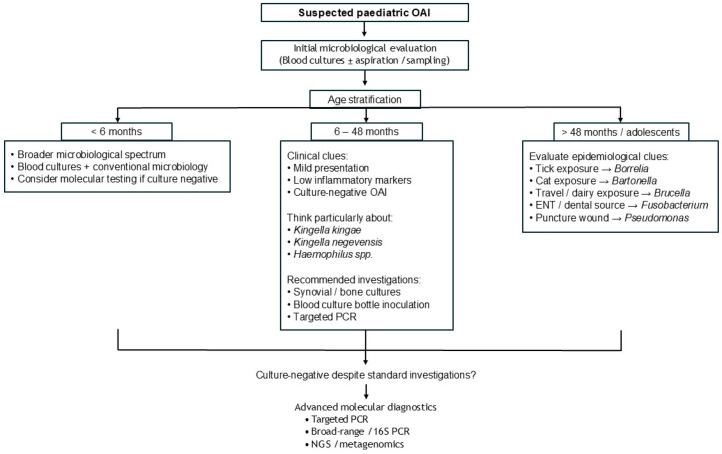
Suggested diagnostic approach for children presenting with atypical or culture-negative paediatric osteoarticular infections, integrating age-related epidemiology, clinical clues, exposure history and microbiological investigations.

Figure 1 summarises a suggested diagnostic approach for children presenting with atypical or culture-negative paediatric OAIs.

## 4. Hacek Group Microorganisms

The acronym HACEK refers to a group of fastidious Gram-negative microorganisms, including *Haemophilus* spp., *Aggregatibacter actinomycetemcomitans* (formerly *Actinobacillus actinomycetemcomitans*), *Cardiobacterium hominis*, *Eikenella corrodens* and *Kingella kingae*. HACEK microorganisms grow slowly in standard blood cultures, often requiring extended incubation [12]. Usually found in the oropharynx, they are primarily known for causing infective endocarditis, but they can also lead to OAIs. In the context of this review, pathogens were included not solely according to their rarity, but based on one or more predefined characteristics, including fastidious microbiological behaviour, association with culture-negative infections, emerging recognition through molecular diagnostics, exposure-related epidemiology, or atypical clinical presentation in paediatric OAIs. Consequently, pathogen selection was based primarily on diagnostic and clinical relevance rather than exclusively on incidence.

### 4.1. Kingella kingae

OAIs caused by *K. kingae* are the prototypical example of fastidious, frequently culture-negative, Gram-negative musculoskeletal infections in young children. Since the 1980s, the reported number of cases of *K. kingae* OAIs has markedly increased, mainly due to the use of molecular identification methods [13,14,15,16,17]. *K. kingae* is thus now regarded as the leading bacterial cause of paediatric OAIs, especially in children under 48 months old [18,19]. In addition to classic acute haematogenous osteomyelitis and septic arthritis, this pathogen can cause atypical OAIs, including spondylodiscitis [20,21,22,23,24,25], subacute osteomyelitis [26,27,28], pyomyositis [29], bursitis [30] and tendon sheath infections [31,32,33].

*K. kingae* septic arthritis generally involves large weight-bearing joints such as hips, knees, ankles, shoulders or elbows [14,15,18,19,34,35,36]. Atypical joints, such as small metacarpophalangeal and metatarsophalangeal joints and sternoclavicular, acromioclavicular or tarsal joints, are over-represented in cases of *K. kingae* arthritis, compared with paediatric septic arthritis overall [13,14,15,18,19,34,35,36]. The anatomical sites involved in paediatric patients with *K. kingae* osteomyelitis include long bones such as the femur, tibia, humerus, radius and ulna [14,15,18,19,34,37]. A dedicated case series focusing on *K. kingae*-induced septic arthritis of the knee in young children emphasised its relatively mild clinical presentation and modest biological inflammatory response [38]. Nevertheless, any bone rarely infected by other pathogens, such as the calcaneum, talus, sternum or clavicle, can also be affected by *K. kingae* osteomyelitis [18,19,34,35,36]. The onset of a *K. kingae* OAI is generally insidious, and the disease is frequently diagnosed after a considerable delay, at a time when bone lesions are already subacute in character and can present as lytic lesions [31,32]. Finally, the clinical course is usually better for children with an OAI due to *K. kingae* rather than other typical pathogens, as evidenced by shorter hospitalisations and fewer adverse events [18,19,34,39].

### 4.2. Kingella negevensis

*K. negevensis* is a newly described Gram-negative bacterium closely related to *K. kingae*. Like *K*. *kingae*, *K*. *negevensis* can be isolated from the oropharynx of young children, and, in some cases, it is probably misidentified as *K*. *kingae*. Indeed, *K*. *negevensis* produces many of the same virulence factors as *K*. *kingae*, including a polysaccharide capsule, a secreted exopolysaccharide, a Knh-like trimeric autotransporter and type IV pili, suggesting that *K*. *negevensis* has significant pathogenic potential and can cause OAIs [40,41].

### 4.3. Non-Type-B Haemophilus influenzae, Unencapsulated H. influenzae and H. parainfluenzae

In the post-vaccination era, *Haemophilus* spp. rarely cause OAIs, with *H. influenzae* type b (Hib) now rare in countries with widespread immunisation programmes. However, some non-typeable *H. influenzae* (NTHi) and other encapsulated strains (particularly serotypes a, f and e) that are not targeted by immunisation continue to cause OAIs [42,43,44,45]. At Texas Children’s Hospital, from 2011 to 2018, non-typeable strains accounted for 39.3% of invasive *H. influenzae* infections [46,47,48,49], and type a accounted for 29.5% [50]. *H. influenzae* type a is the most common serotype, although types d, e and f are also reported. Another *Haemophilus* species, *Haemophilus parainfluenzae*, is an extremely rare cause of paediatric OAIs, with only a handful of cases reported in the literature [51,52,53].

These OAIs typically affect children younger than 4 years, with the highest prevalence between 7 and 24 months old [54]; septic arthritis is the most common manifestation. Indeed, a recent systematic review of the literature documented just 21 cases of acute septic arthritis due to non-type-b *H. influenzae* [46]. As was often the case with OAIs due to Hib, children tended to be under 2 years old and present with a monoarticular acute septic arthritis, usually affecting the knee [46]. Acute haematogenous osteomyelitis is much less common with non-type-b *H. influenzae*, and it may be concurrent with acute septic arthritis in up to 40% of cases [46].

Unencapsulated strains of *H. influenzae* (non-typeable *H. influenzae)* appear to be much rarer causes of OAIs than non-type-b *H. influenzae*, with only scattered reports in the literature, primarily describing acute septic arthritis [55]. From a practical viewpoint, given their rarity and potentially lower virulence, isolating an unencapsulated strain of *H. influenzae* should prompt an immunological evaluation of the child.

### 4.4. Aggregatibacter actinomycetemcomitans

*Aggregatibacter actinomycetemcomitans* is another member of the HACEK group. It primarily colonises the human oral cavity and is associated with aggressive periodontitis. It can also cause serious extraoral infections, including rare cases of OAIs like osteomyelitis and septic arthritis. Indeed, it has been implicated in osteomyelitis and epidural abscesses in children and adults [56], demonstrating that even slow-growing, Gram-negative microorganisms should be considered in the differential diagnosis of culture-negative OAIs, and emphasising the importance of using tissue cultures and NAAA technologies [57].

## 5. Emerging, Exposure-Related and Rare Pathogens

### 5.1. Emerging Pathogens

#### 5.1.1. *Neisseria meningitidis*

While mostly known for causing meningitis, *N. meningitidis* can also cause osteoarticular disorders, such as septic arthritis, reactive arthritis and osteomyelitis. Arthritis can occur as a primary, isolated infection or can be secondary to sepsis. Joint arthritis may complicate acute meningococcal disease in 5–10% of cases [58,59,60] and commonly affects the knees and hips. Primary septic arthritis due to *N. meningitidis* without accompanying meningitis remains extremely rare and tends to be monoarticular [61,62,63]; it affects the larger joints in patients of all ages [58,59]. Primary meningococcal arthritis has been reported with serotypes W-135, B and C [58]. Alongside septic arthritis directly due to the pathogen, an immune-mediated arthritis may also develop, often appearing later in the disease course [58,60,64] and often being polyarthritic [60]. Given the ease with which this microorganism can be treated with antimicrobial therapy, its relative contribution to septic arthritis is probably underestimated, and some cases of immune-mediated arthritis probably involve eradicated septic arthritis. Acute haematogenous osteomyelitis due to *N. meningitidis* is less common than primary meningococcal arthritis, likely accounting for fewer than 2% of all cases of paediatric osteomyelitis [65,66]. In otherwise healthy children, it has been reported in the tibia [67], fibula [68], acromion [69], lumbar vertebrae [70] and humerus [71]. Serogroup B *N. meningitidis* (for which no vaccine existed until recently) predominates in the literature [67], although serogroups C and Y have also been described [67,68,69,70,71].

#### 5.1.2. *Fusobacterium* spp.

*Fusobacterium* spp. are Gram-negative, anaerobic bacilli that are part of normal oral and gastrointestinal flora; they are usually opportunistic pathogens. OAIs due to anaerobic pathogens are rare, and, when present, *Fusobacterium* spp. are likely their most common cause in otherwise healthy children. *Fusobacterium nucleatum* and *Fusobacterium necrophorum* are the most frequently reported in the literature, although other species, such as *Fusobacterium fusiforme*, have been reported as well [72]. *Fusobacterium* spp. are the most common aetiological agents of Lemierre’s syndrome, which is characterised by bacteraemia involving anaerobic organisms (indeed, usually *Fusobacterium* spp.), internal jugular vein thrombosis, and a metastatic, suppurative spread of infection (abscess formation in various locations) [73]. Many reported cases of OAIs involving *Fusobacterium* spp. are associated with anaerobic sources of infection such as sinus disease, dental abscesses, mastoiditis or dog bites [74]. These infections are often polymicrobial [74], but an isolated disease, without the risk factors for an anaerobic source of infection, may also be observed, especially in children. Thus, *Fusobacterium* spp. may be present because of a metastatic spread of infection with acute septic arthritis [75,76] or acute haematogenous osteomyelitis [73,76]. Acute septic arthritis due to *Fusobacterium* spp. has been reported in hips [77,78,79] and knees [80], and acute haematogenous osteomyelitis has been reported in the pelvic region [81], hips [82] and femurs [83] without the presence of Lemierre’s syndrome or risk factors for anaerobic infection. *Fusobacterium* spp. have also been identified as the causative pathogens in cases of subacute osteomyelitis and pelvic OAIs with Brodie’s abscess in children [84,85]. Interestingly, patients with OAIs due to *Fusobacterium* spp. are often older than those with OAIs due to more typical organisms. Of the 23 cases of OAI involving *Fusobacterium* spp. identified in a narrative review, 19 patients (83%) were over 8 years old, suggesting that OAIs due to *Fusobacterium* spp. tend to occur in older children [86]. These cases are diagnostically challenging, as anaerobic cultures require specific conditions and results are frequently delayed or negative.

### 5.2. Exposure-Related Pathogens

#### 5.2.1. *Borrelia burgdorferi*

*Lyme borreliosis* is a zoonotic disease caused by *Borrelia burgdorferi*, a member of the Spirochaetaceae family. *Borrelia burgdorferi*’s vectors are ticks of the Ixodes genus, which transmit the pathogen to human hosts while feeding on their blood. The typical clinical picture of Lyme disease includes symptoms involving the skin, joints, nervous system and, more rarely, the heart and eyes [87,88]. Lyme disease has three main stages: early localised, early disseminated and late disseminated infections. Musculoskeletal symptoms are common, but arthritis—characterised by swelling and pain in one or more large joints—is the main sign of *Lyme borreliosis*. Patients with confirmed borreliosis may also experience tendon, muscle or bone pain [89,90]. Joint involvement is typically asymmetrical and mostly in large joints, particularly the knees, but small joints may also be affected as components of oligoarthritis. Apart from knees, Lyme arthritis commonly also affects shoulder, elbow, wrist and ankle joints [87,89,90,91]. Lyme arthritis results from a strong inflammatory response (not from spirochete toxins) that causes subsequent host-driven aggrecan degradation and leads to cartilage damage and severe joint injury [87]. In the early localised stage of erythema migrans, almost half of patients experience migratory muscle and joint pain. The time between a tick bite and the onset of Lyme arthritis can vary greatly, ranging from a few days to several months [92,93]. If present for longer durations, Lyme arthritis can lead to the erosion and destruction of joint structures.

#### 5.2.2. *Brucella* spp.

Brucellosis is a zoonotic infection caused by species of the *Brucella* genus. *Brucella* are small, aerobic, intracellular coccobacilli that preferentially localise in the reproductive organs of host animals (mainly ruminants), causing abortions and infertility in infected animals. The bacteria are transmitted from animals to humans through the ingestion of infected food (mainly unpasteurised dairy products), direct contact with infected animal products or viscera, or the inhalation of aerosols [94,95].

Brucellosis is primarily found among children living in endemic areas, but the disease has also been reported among returning travellers or individuals residing in regions bordering endemic areas [94,96]. Travel and globalisation both increase the likelihood that patients will present with a potential *Brucella* infection that has moved from an endemic to a non-endemic area. Alongside the classic clinical picture, which combines several non-specific systemic features (fever, fatigue, weight loss, hepatosplenomegaly), *Brucella* frequently presents together with arthritis or arthralgias (up to 40–80% in some series) that can mimic the presence of septic arthritis (with invasion of the organism into the joint space) [97,98]. However, arthritis is usually reactive in nature [99], polyarticular, additive and non-migratory, typically involving the sacroiliac region, knees or hips [94,95,97,100,101]. Alongside reactive arthritis, nearly 10% of individuals infected with *Brucella* spp. and presenting with osteoarticular complaints have their bacterial growth confirmed by synovial fluid culture [99]. However, this microorganism’s relatively fastidious nature may preclude its more frequent isolation in culture, leading to an underestimation of the true incidence of acute septic arthritis occurring with this pathogen [102]. Knees and hips are the joints most frequently affected by culture-proven acute septic arthritis due to *Brucella* spp. [103]. Though less commonly reported than acute septic arthritis, acute haematogenous osteomyelitis may also be caused by *Brucella* spp., with cases involving osteomyelitis of the calcaneus, the sternum and olecranon, and the femur described in the literature [100,103,104,105].

The direct inoculation of synovial fluid into blood culture bottles may improve culture yield and increase the isolation rates of *Brucella* spp. [102]. Real-time qPCR has emerged as a rapid, sensitive and specific tool for diagnosing brucellosis, overcoming many of the limitations of traditional culture and serological methods. While bacterial culture remains the gold standard assay, it is time-consuming and not very sensitive, making qPCR the preferred alternative for quick, genus-level identification of *Brucella* [106,107].

#### 5.2.3. *Salmonella* spp.

OAIs caused by *Salmonella* spp. are rare but serious, usually manifesting as osteomyelitis or septic arthritis, often after bacteraemia in immunocompromised individuals or those with sickle cell disease. Non-typhoidal *Salmonella* can also cause invasive infections, but there are no risk factors in young children, especially under 1 year old [108]; a Taiwanese series found that over 60% of bacteraemia cases occurred in this age group [108]. However, the distribution of non-typhoidal *Salmonella osteomyelitis* in healthy children does not reveal a predominance of involvement among infants. Indeed, one series demonstrated that among the 46 cases of non-typhoidal *Salmonella osteomyelitis* reviewed in healthy children, only 19% were infants, with a median age of 9 years old and male predominance (61%) [109]. The most common serotypes were *S. Enteritidis* and *S. Typhimurium*. Complications were frequent and occurred in 41% of subjects, including relapses, multifocal disease or suppurative issues [109].

Acute septic arthritis with non-typhoidal *Salmonella* is rare. Sirinavin et al. described 82 invasive infections involving non-typhoidal *Salmonella* in immunocompetent children, only 2 (2.4%) of which involved acute septic arthritis [110], and Punpanich et al. reported that just 1.3% of 226 children with a culture-confirmed, invasive non-typhoidal *Salmonella* infection developed acute septic arthritis [111]. Acute septic arthritis due to non-typhoidal *Salmonella* has been reported in otherwise healthy children in elbows, sacroiliac joints, knees, metacarpophalangeal joints and shoulders [112,113,114,115,116]. Acute septic arthritis due to non-typhoidal *Salmonella* can be difficult to distinguish from reactive arthritis associated with gastrointestinal *Salmonella* infections [117,118]. Reactive arthritis due to *Salmonella* appears to have a slight female bias and is more common in adults than children [119]. Its incidence varied from 1.2–29.0% [119], presenting as either mono- or oligoarticular, and most commonly affecting the knee [117].

### 5.3. Rare, Exceptional and Diagnostically Challenging Pathogens

#### 5.3.1. *Actinomyces* spp.

*Actinomyces* spp. are anaerobic Gram-positive bacteria that usually colonise the mouth, gut or genital tract. Infection occurs when they invade tissue during dental work, through poor oral hygiene or due to trauma. *Actinomyces* is therefore another anaerobic organism occasionally associated with OAIs. *Actinomyces israelii* is the most commonly reported species, though *Actinomyces odontolyticus*, *Actinomyces viscosus*, *Actinomyces turicensis* and *Actinomyces naeslundii* have also been mentioned [120,121,122]. *Actinomyces* OAIs are chronic in character and often follow a very indolent course, mimicking tumours or tuberculosis [121]. A systematic review of osteomyelitis cases due to *Actinomyces* spp. demonstrated a median time to presentation of 3 months, with three cases only being recognised after 2 years [121]. *Actinomyces* spp. are common flora in the oral cavity, which explains why over half of all *Actinomyces* infections involve the cervicofacial region [73]. Because of this tendency, the mandible is the most frequently affected bone in cases of osteomyelitis, before spondylitis of the C1 and C2 vertebrae [120,123]. Osteomyelitis of the iliac bone has also been encountered due to the disease spreading from the pelvis. Culture attempts are essential, but given the microorganism’s fastidious nature, NAAAs or a histological analysis may be required for diagnosis [120,121,123]. Histopathological analysis of *Actinomyces* infections often demonstrates infiltrative spread across tissue and fascial planes, sinus tract formation, sulphur granules, and Gram-positive branching organisms with acute-angle branching [86].

#### 5.3.2. Other Rare Pathogens

Group C and G *Streptococci* (GCGS) OAIs in children are uncommon compared to other pathogens, with these microorganisms predominantly affecting elderly adults rather than paediatric populations. GCGS OAIs in humans generally produce a less acute clinical presentation than Group A *Streptococcus* [124]. Indeed, many GCGS in humans belong to the *Streptococcus dysgalactiae* subspecies *equisimilis* (SDSE), which shares many virulence factors with *S. pyogenes* through horizontal gene transfer [125,126,127]. Thus, GCGS have not been listed among the major pathogens in recent large prospective studies of paediatric OAIs [128,129].

A few cases of OAIs due to GCGS in otherwise healthy children have nevertheless been reported in the literature, with just one case of acute haematogenous osteomyelitis and six cases of acute septic arthritis [130,131,132,133,134]. Many laboratories are unable to identify isolates down to the species level (e.g., *Streptococcus dysgalactiae*, *S. equi*) and instead use the less specific Lancefield classification. Although data are too sparse to ascribe virulence differences to different species, and although all their isolates appear susceptible to penicillin, their susceptibility to non-beta-lactam agents (as may be required in penicillin-allergic patients) is less predictable and may vary by species or subspecies [91].

*Staphylococcus caprae* is a coagulase-negative staphylococcus rarely implicated in bone infections. Bone and joint infections due to *S. caprae* have been reported, but fortunately they remain rare [135,136,137,138]. Most *S. caprae* OAIs are the result of infected orthopaedic devices, especially infected joint prostheses and internal osteosynthesis devices [139,140,141,142]. Only very rare cases of primary *S. caprae* OAIs have been described, including osteitis [135,136], subacute osteomyelitis [138], arthritis [136] and spondylodiscitis [137,143].

*Bartonella henselae* OAIs caused by this Gram-negative bacterium are rare and atypical complications of cat scratch disease (CSD), which usually results from a scratch or bite from a young cat [144,145]. While typically presenting as a self-limiting lymphadenopathy, it can also cause osteomyelitis or septic arthritis. Even though cases of septic arthritis have been reported with this germ [146], OAIs due to *B. henselae* almost exclusively present as acute haematogenous osteomyelitis and demonstrate a strong tendency to affect the axial skeleton and pelvis, with nearly 75% of published cases localised to these areas [147,148]. Although osteomyelitis is an uncommon manifestation of CSD, it is a significant atypical presentation of paediatric bartonellosis and may be under-recognised in children with subacute axial or pelvic bone lesions. Furthermore, *B. henselae* has been increasingly recognised as a cause of atypical subacute osteomyelitis in children and adolescents [28]. Lymphadenopathy may be seen in approximately 25% of patients with CSD, which is not typical in cases of OAI [147]. Acute and convalescent serological studies and PCR testing may assist in diagnoses, given the microorganism’s fastidious nature [147,148,149]. In a similar vein, Vazquez et al. reported on a series of paediatric patients presenting with OAIs with atypical features, thus emphasising the need to use systematic serological and PCR testing appropriate to the epidemiological context [150]. One unknown remains regarding the treatment of these OAIs: given that antibiotic treatment of *Bartonella* lymphadenitis has not been shown to significantly improve long-term outcomes, it is unclear to what extent the treatment of OAIs hastens disease resolution [151]. However, given the lack of observational data for OAI disease processes, as well as the risk of morbidity in childhood OAIs, many authors and clinicians have continued to use antibiotic treatments.

*Bordetella holmesii* is a Gram-negative, rod-shaped bacterium of the Bordetella genus. Less well-known than its more famous relative *Bordetella pertussis*, this pathogen is considered to be an emerging bloodstream pathogen, more likely to affect immunocompromised children, such as asplenic patients [152]. However, native septic arthritis due to *B. holmesii* has been reported in adolescents, particularly in the setting of underlying haematological or immune conditions [153].

Similarly, *Moraxella lacunata* has also been identified as the causative agent of subacute osteomyelitis in a child, highlighting that even unusual members of the oropharyngeal microbiota can produce bone infections. *Moraxella* and *Kingella* are closely related Gram-negative bacteria in the *Neisseriaceae* family, often appearing as coccobacilli in pairs or short chains. Both are fastidious, non-motile, oxidase-positive and catalase-negative, and they commonly inhabit the human respiratory tract as opportunistic pathogens that can cause similar infections, such as endocarditis and OAIs.

*Morganella morganii* is a Gram-negative, facultatively anaerobic bacterium commonly found in the human intestinal tract and the environment, and as a commensal in many animals. It is typically considered an opportunistic pathogen and a significant cause of nosocomial infections. *M. morganii* has been reported as the causative agent of a Brodie’s abscess in a child’s talus, a presentation that highlights the expanding bacteriological spectrum of subacute OAIs [154].

*Burkholderia pseudomallei* can rarely cause isolated OAIs in otherwise healthy children, particularly in Southeast Asia and northern Australia [155]. The disease is more common in lower limbs, potentially because inoculation often occurs from walking barefoot through infected soil or water [155].

*Pseudomonas aeruginosa* is a common, highly adaptable, Gram-negative bacterium found in soil and water. It is usually implicated in infections following puncture wounds to the foot, manifesting with a sweetish odour and a greenish discolouration [156,157]. However, this pathogen can also be a rare cause of OAIs in the absence of trauma, occasionally as tibial osteomyelitis, pelvic osteomyelitis (potentially owing to its occasional presence in faecal flora) or, given its tendency to cause otic infections, as osteomyelitis of the skull associated with complications arising from mastoiditis [158,159,160].

## 6. Conclusions

Advances in nucleic acid amplification assay technologies have revolutionised diagnostics for infectious diseases. These advances have enabled better identification and resistance profiling of the causative pathogens responsible for paediatric osteoarticular infections (OAIs). It has also now been increasingly observed that otherwise healthy children may present with OAIs secondary to unanticipated pathogens. Although culture-negative results may become less frequent with the broader use of molecular diagnostics, clinicians are likely to encounter an expanding spectrum of uncommon, fastidious and emerging bacterial pathogens. Due to their infrequent occurrence and limited clinical experience with these atypical pathogens, heightened awareness is essential for the accurate identification and effective management of such infections. For paediatric orthopaedic surgeons, this evolving microbiological landscape also reinforces the need to consider uncommon pathogens in culture-negative, subacute OAIs, including those at atypical sites or resulting from atypical exposures.

## Figures and Tables

**Table 1:** Microbiological and clinical profiles of fastidious, emerging, exposure-related and rare bacterial pathogens causing paediatric OAIs, grouped according to their microbiological, epidemiological and clinical context.

Pathogen	Gram/ Microbiological Category	Reservoir or Exposure	Paediatric Clinical Context	Main OAI Phenotype	Reported or Predominant Sites	Diagnostic Approach	Key Clinical Message
*Kingella kingae*	Gram-negative β-haemolytic coccobacillus; Neisseriaceae family; member of the HACEK group.	Oropharyngeal carriage; often associated with recent upper respiratory tract infection or stomatitis.	Children 6–48 months old; frequently mild or afebrile presentation, with low-to-moderate inflammatory markers.	Septic arthritis; also osteomyelitis, spondylodiscitis, subacute osteomyelitis, and rarer soft-tissue, bursal or tendon-sheath infections.	Knee, hip, ankle, shoulder and elbow; may also involve small hand/foot joints, calcaneus, talus, clavicle, sternum and spine.	Culture and Gram stain often negative; targeted PCR from synovial fluid, bone or abscess material; throat swab PCR may provide supporting evidence.	Molecular-era prototype; leading cause of culture-negative OAI under 4 years old.
*Kingella negevensis*	Gram-negative coccobacillus; Neisseriaceae family; closely related to *K. kingae*.	Oropharyngeal carriage in young children.	Young children; probably under-recognised because of possible misidentification as *K. kingae*.	Possible OAI pathogen reported in rare cases; independent clinical phenotype remains poorly defined.	No reproducible anatomical tropism established; sites should not be extrapolated from *K. kingae*.	Species-level identification is required, as some *Kingella*-targeted molecular assays may not distinguish *K. negevensis* from *K. kingae*.	Emerging *Kingella* species; should not be presented as an established major paediatric OAI pathogen.
Non-type-b/non-typeable *H. influenzae* and *H. parainfluenzae*	Gram-negative pleomorphic coccobacilli; fastidious respiratory organisms.	Nasopharyngeal and upper respiratory tract carriage.	Exceptionally rare in immunocompetent children.	Mainly septic arthritis; osteomyelitis is less frequent; possible combined arthritis/osteomyelitis.	Knee and other large joints; hip involvement reported.	Culture/PCR; species confirmation and capsular typing are essential.	Important mainly in the post-Hib vaccination era; distinguish encapsulated non-type-b strains from non-typeable isolates.
*Aggregatibacter actinomycetemcomitans*	Gram-negative coccobacillus; facultative anaerobe; member of the HACEK group.	Oral cavity; aggressive periodontitis; possible haematogenous dissemination from an oral source.	Very rare; may present with muted systemic inflammation and delayed diagnosis.	Subacute/chronic osteomyelitis; rare septic arthritis; vertebral osteomyelitis, spondylodiscitis and epidural abscess reported.	Spine/epidural space and distal skeletal sites; no consistent paediatric anatomical tropism.	Deep tissue sampling; prolonged incubation, MALDI-TOF, 16S rRNA sequencing/PCR or targeted molecular identification.	Useful oral HACEK organism illustrating culture-negative or delayed-diagnosis OAI.
*Neisseria meningitidis*	Encapsulated Gram-negative diplococcus; Neisseriaceae family; serogroup-defined invasive pathogen.	Nasopharyngeal carriage; respiratory transmission; invasive meningococcal disease may be present or absent.	Children and adolescents; may occur during invasive meningococcal disease or as isolated septic arthritis.	Primary meningococcal septic arthritis; septic arthritis complicating invasive meningococcal disease; delayed immune-mediated/reactive arthritis; osteomyelitis is exceptional.	Large joints, especially knees and hips; osteomyelitis reported only rarely in long bones or vertebral sites.	Blood/synovial culture and/or PCR; serogroup identification; timing and synovial findings help distinguish septic from immune-mediated arthritis.	Rare but important cause of paediatric septic arthritis; distinguish primary infection from post-meningococcal immune-mediated arthritis.
*Borrelia burgdorferi*	Spirochete; not reliably classified using a routine Gram stain; vector-borne zoonotic pathogen.	Ixodes tick exposure; residence in or travel to Lyme-endemic regions.	Children and adolescents in endemic areas; often subacute, intermittent or recurrent mono-/oligoarticular swelling.	Lyme arthritis; subacute inflammatory mono-/oligoarthritis rather than typical acute pyogenic septic arthritis.	Predominantly knees; less commonly ankles, elbows, shoulders or wrists.	Two-tier serology is central; synovial fluid PCR may be used as an adjunct in selected seropositive cases when diagnostic uncertainty persists.	Frame separately from pyogenic septic arthritis; drainage only if pyogenic infection cannot be excluded.
*Brucella* spp.	Small Gram-negative facultative intracellular coccobacilli; zoonotic pathogen.	Unpasteurised dairy products, livestock exposure, endemic areas or travel.	Children from, living in or returning from endemic regions.	Arthralgia, peripheral arthritis, sacroiliitis, spondylitis/ spondylodiscitis and osteomyelitis.	Sacroiliac region, hips, knees and spine; occasional focal bone involvement.	Serology, blood/synovial culture and PCR; alert the laboratory because cultures may require prolonged incubation.	Exposure and travel history are central; distinguish inflammatory arthropathy from culture- or PCR-confirmed infection.
*Salmonella* spp.	Gram-negative facultative intracellular bacilli; mainly non-typhoidal Salmonella.	Gastrointestinal tract; contaminated food or reptile exposure; haematogenous dissemination after bacteraemia.	Typically associated with sickle cell disease, haemoglobinopathies or immunocompromised status, but may occur in otherwise healthy children.	Osteomyelitis is more common than septic arthritis; may be multifocal, complicated or relapsing.	Long bones, pelvis and spine; septic arthritis reported in knee, hip, shoulder, elbow, sacroiliac and small joints.	Blood, bone or synovial culture; susceptibility testing required; distinguish from reactive arthritis after gastroenteritis.	Host- and exposure-related Gram-negative OAI; emphasise complications and relapse.
*Fusobacterium* spp.	Gram-negative anaerobic bacilli; mainly *F. necrophorum* and *F. nucleatum*.	Oral, ENT and gastrointestinal flora; dental infection, sinusitis, pharyngitis, mastoiditis; Lemierre’s syndrome.	Mostly older children and adolescents.	Septic arthritis, acute haematogenous osteomyelitis, pelvic OAI, Brodie’s abscess; possible metastatic infection in Lemierre’s syndrome.	Hips, knees, pelvis and femurs; occasionally multifocal.	Anaerobic cultures require specific handling and may be delayed or negative; 16S rRNA PCR can be useful.	Adolescent age, ENT/dental source, pelvic or hip involvement, and anaerobic sampling are key diagnostic triggers.
*Actinomyces* spp.	Gram-positive anaerobic branching rods; slow-growing commensal organisms.	Oral, gastrointestinal and genital flora; dental disease, trauma and mucosal disruption.	Rare in children; usually chronic, indolent presentation.	Chronic osteomyelitis with tissue-plane invasion or sinus tract formation.	Mandible and cervicofacial skeleton; C1–C2 spine; pelvis.	Prolonged anaerobic culture, histology, sulphur granules and 16S rRNA PCR.	Chronic invasive infection that may mimic malignancy, tuberculosis or chronic nonbacterial osteomyelitis.
Group C/G *streptococci*, including *Streptococcus dysgalactiae* subsp. *equisimilis*	Gram-positive beta-haemolytic cocci.	Skin and upper respiratory tract.	Rare in otherwise healthy children; more common in adults.	Septic arthritis more often than osteomyelitis.	Elbows, knees and other large joints.	Usually standard culture-positive; species-level identification may be absent.	Uncommon but usually culture-positive pyogenic pathogen; best framed separately from fastidious or molecular-era organisms.
*Staphylococcus caprae*	Gram-positive coagulase-negative Staphylococcus.	Skin flora; historically associated with goats; orthopaedic implants/devices.	Mostly device- or implant-associated; primary paediatric OAI is exceptional.	Osteitis, subacute osteomyelitis, arthritis, spondylodiscitis.	Varied; implant-associated sites predominate.	Culture; clinical correlation needed to avoid dismissal as a contaminant.	Unusual coagulase-negative Staphylococcus; clinical relevance should be considered only when isolation is microbiologically credible and supported by concordant clinical or radiological findings.
*Bartonella henselae*	Gram-negative fastidious intracellular bacillus.	Cat scratch or bite; kitten exposure.	School-age children/ adolescents; subacute presentation.	Subacute osteomyelitis; rare septic arthritis; atypical cat scratch disease.	Pelvis and axial skeleton predominate; vertebrae and acetabulum reported.	Serology, tissue PCR or molecular testing; lymphadenopathy may be absent in isolated OAI presentations.	Bone involvement is uncommon in typical cat scratch disease but should be considered in atypical paediatric bartonellosis, particularly with pelvic or axial lesions.
*Bordetella holmesii*	Gram-negative rod/coccobacillus.	Respiratory tract; bloodstream pathogen.	Asplenia, haemoglobinopathy or immunocompromised status; exceptional reports in adolescents.	Septic arthritis.	Knee reported.	Culture with molecular/species-level identification.	Very rare emerging bloodstream pathogen; consider only in a compatible host or culture-confirmed septic arthritis.
*Moraxella lacunata*	Gram-negative diplobacillus/coccobacillus; fastidious Neisseriaceae-related organism.	Upper respiratory tract and ocular flora.	Very rare paediatric reports.	Subacute osteomyelitis.	Limited data; case-based.	Culture or molecular identification.	Exceptional case-based pathogen; include only as a rare fastidious Gram-negative cause when supported by paediatric reports.
*Morganella morganii*	Gram-negative facultative anaerobic bacillus; Enterobacterales.	Gastrointestinal tract, environment, animal commensal flora.	Very rare in healthy children.	Brodie’s abscess, subacute osteomyelitis.	Talus reported in case-based literature.	Standard culture; susceptibility testing is important.	Exceptional Enterobacterales-associated OAI; mainly relevant when culture-confirmed and supported by concordant imaging.
*Burkholderia pseudomallei*	Gram-negative bacillus.	Soil and surface water; endemic to Southeast Asia and northern Australia.	Residence or travel in endemic areas; inoculation through skin, often barefoot exposure.	Osteomyelitis, septic arthritis, melioidosis-related skeletal infection.	Lower limb and foot/metatarsal involvement reported.	Culture; requires epidemiological suspicion and laboratory safety awareness.	Geographically driven pathogen; travel, residence in endemic areas and soil/water exposure are key diagnostic triggers.
*Pseudomonas aeruginosa*	Gram-negative aerobic bacillus.	Soil/water; puncture wounds; otitis/mastoiditis; hospital environment.	Usually traumatic inoculation or puncture wound; rare non-traumatic cases in healthy children.	Foot osteomyelitis after puncture wound; tibial, pelvic, or skull-/mastoid-related osteomyelitis.	Foot, tibia, pelvis and skull base/mastoid region.	Culture; exposure history; consider after plantar puncture through a shoe or an otogenic source.	Exposure-driven pathogen; consider after plantar puncture wounds, otogenic infection or healthcare-associated exposure.

Abbreviations: ENT = ear, nose and throat; HACEK = *Haemophilus* spp., *Aggregatibacter* spp., *Cardiobacterium hominis*, *Eikenella corrodens* and *Kingella* spp.; Hib = Haemophilus influenzae type b; MALDI-TOF = matrix-assisted laser desorption/ionisation time-of-flight mass spectrometry; OAI = osteoarticular infection; PCR = polymerase chain reaction; rRNA = ribosomal RNA.

## Data Availability

Data sharing is not applicable to this article, as no datasets were generated or analyzed during the preparation of this review.

## Abbreviations

The following abbreviations are used in this manuscript:

OAIs	Osteoarticular infections
PCR	Polymerase chain reaction
NAAAs	Nucleic acid amplification assays
Hib	H. influenzae type b
GCGS	Group C and G Streptococci
SDSE	Streptococcus dysgalactiae subspecies equisimilis
CSD	Cat scratch disease
